# Impact of Obesity on the Bioavailability of Peginterferon-α2a and Ribavirin and Treatment Outcome for Chronic Hepatitis C Genotype 2 or 3

**DOI:** 10.1371/journal.pone.0037521

**Published:** 2012-05-24

**Authors:** Åsa Alsiö, Karolina Rembeck, Galia Askarieh, Peer Brehm Christensen, Martti Färkkilä, Nina Langeland, Mads Rauning Buhl, Court Pedersen, Kristine Mørch, Bart L. Haagmans, Salmir Nasic, Johan Westin, Kristoffer Hellstrand, Gunnar Norkrans, Martin Lagging

**Affiliations:** 1 Department of Infectious Diseases/Virology, Institute of Biomedicine, University of Gothenburg, Gothenburg, Sweden; 2 Department of Infectious Diseases, University of Southern Denmark, Odense, Denmark; 3 Department of Gastroenterology, Helsinki University, Helsinki, Finland; 4 Department of Medicine, Haukeland University Hospital and Institute of Medicine, University of Bergen, Bergen, Norway; 5 Department of Infectious Diseases, Aarhus University, Aarhus, Denmark; 6 Department of Virology, Erasmus MC, Rotterdam, The Netherlands; 7 Department of Research and Development/Statistics, Skaraborg Hospital, Skövde, Sweden; Saint Louis University, United States of America

## Abstract

**Background and Aims:**

Having a body mass index above or equal to 30 kg/m^2^ in conjunction with chronic hepatitis C virus infection is associated with non-responsiveness to treatment with interferon and ribavirin, but details regarding the mechanisms whereby obesity reduces the efficacy of therapy remain unclear.

**Methods:**

This study evaluated impact of obesity on outcome as well as interferon and ribavirin concentrations following standard-of-care fixed dosing with peginterferon-α2a 180 µg once weekly and ribavirin 800 mg daily among 303 HCV genotype 2/3-infected patients enrolled in the per-protocol analysis of a recently completed phase III trial (NORDynamIC).

**Results:**

Patients with BMI ≥30 kg/m^2^ showed poorer outcome following 24 weeks of therapy (SVR 62% vs. 89% for BMI ≥30 vs. <30; P = 0.006) along with significantly higher steatosis grade (P = 0.002), HOMA-IR (P<0.0001), triglyceride levels (P = 0.0002), and baseline viral load (P = 0.028). Obesity was also significantly associated with lower plasma interferon concentrations on days 3, 7, and 29 (P = 0.02, P = 0.0017, and P<0.0001, respectively) and lower plasma ribavirin concentrations day 29 (P = 0.025), and lower concentration of interferon in turn was associated with a poorer first phase reduction in HCV RNA (P<0.0001). In multivariate analysis, ribavirin concentrations week 12, interferon concentrations day 29, and baseline HCV RNA levels were independent predictors of achieving SVR among patients treated for 24 weeks (n = 140).

**Conclusions:**

Reduced bioavailability of interferon and ribavirin along with higher baseline viral load are dominant risk factors for treatment failure in obese patients with chronic hepatitis C.

## Introduction

Hepatitis C virus (HCV) is the foremost cause of parenterally transmitted hepatitis [1], [2], and chronic infection is associated with liver fibrosis, cirrhosis, and hepatocellular carcinoma [3], [4]. Treatment with pegylated interferon-α and ribavirin yields sustained viral response (SVR) rates of 50–80% [5], [6], [7], with higher SVR rates seen in patients infected with HCV of genotypes 2 and 3.

Thirty percent of American adults are obese, *i.e.* have a body mass index (BMI) above or equal to 30 kg/m^2^ [8]. High BMI in conjunction with HCV is associated with steatosis in HCV genotype non-3 [9], increased fibrosis progression [10], and non-responsiveness to antiviral treatment [11], [12]. Details regarding the mechanisms whereby obesity reduces the efficacy of combination therapy, however, remain unclear. It has been hypothesized that high BMI induces: (i) the metabolic syndrome leading to insulin resistance, hepatic steatosis, and higher baseline viral load [13], (ii) altered cytokine signaling as manifested by elevated levels of leptin, adiponectin, and resistin [14], and (iii) reduced bioavailability of interferon-α [15]. The aim of this study was to identify independent risk factors for treatment failure among obese patients who participated in a phase III trial (NORDynamIC; n = 303) using standard dosing of peginterferon-α2a and ribavirin in chronic HCV genotype 2 and 3 infection.

## Materials and Methods

### The NORDynamIC trial

Three hundred and eighty-two treatment naïve patients with HCV genotype 2/3 infection were randomized at baseline to either 12 or 24 weeks of combination treatment with peginterferon-α2a 180 µg once weekly and ribavirin 800 mg daily. The first dose of study medication was administered under the supervision of a study nurse, and a patient diary monitored subsequent dosing. Because plasma concentrations of peginterferon-α2a and ribavirin were analyzed and in order to minimize the impact of adherence, patients constituting the per-protocol population (*i.e.* having received at least 80% of the target dose of peginterferon-α2a as well as at least 80% of the target dose of ribavirin for at least 80% of the target treatment duration) were included. Of 303 eligible patients, a body mass index could be calculated for 300, and these latter patients were included in the present study (baseline characteristics in Table 1). Further details regarding this trial are provided elsewhere [16].

**Table 1:** Baseline Characteristics of the Patients Grouped by BMI (Per-Protocol Analysis).

	BMI <30 kg/m^2^	BMI ≥30 kg/m^2^	P
Characteristic	(n = 258)	(n = 42)	
Gender			
Male^a^	158 (61%)	26 (63%)	0.8^d^
Female^a^	100 (39%)	15 (37%)	
Age (years)^b^	41.0	42.7	0.3^e^
Route of transmission^a^			
Intravenous drug use	187 (72%)	32 (73%)	0.75^d^
Transfusion	18 (7%)	4 (10%)	
Health care worker	7 (3%)	0	
Sexual	11 (4%)	1 (2%)	
Unkown	35 (14%)	4 (10%)	
Genotype 2	75 (29%)	15 (37%)	0.24^d^
Genotype 3	184 (71%)	27 (66%)	
Fibrosis Stage (Ishak score 0/1/2/3/4/5/6)	10/34/76/58/35/11/17	0/5/11/14/2/4/2	0.2^d^
Steatosis Grade (0/1/2/3)	95/88/38/20	5/14/12/7	0.002^d^
Log_10_ HCV-RNA (IU/mL)^b^	6.04	6.34	0.028^e^
*IL28B* SNP polymorphism			
(*rs12979860* CC/CT/TT)	111/108/28	18/19/3	0.75^d^
Race^a^ (Caucasian/Asian/Black/Other)	243/6/1/8	42/0/0/0	0.46^d^
Cholesterol (mmol/L)^b^	4.1 (1.1)	4.4 (1.0)	0.9^e^
Triglyceride (mmol/L)^b^	1.0 (0.6)	1.5 (1.0)	0.0002^e^
Systolic Blood Pressure (mm Hg)^b^	126 (15)	138 (20)	0.0004^e^
Diastolic Blood Pressure (mm Hg)^b^	78 (10)	85 (12)	0.002^e^
Alcohol Consumption (units/week)^c^	0.25 (0–18)	1 (0–30)	0.84^e^

a No. (%);

b Mean (SD);

c Median (Range);

d Chi square test,

e Mann-Whitney U-test; two patients were infected with both genotype 2 and 3.

### Classification of response

Patients were classified as achieving SVR if plasma HCV RNA was undetectable (*i.e.* <15 IU/mL) 24 weeks after completion of therapy, as having relapsed if plasma HCV RNA was undetectable at end-of-treatment but detectable 24 weeks after completion of therapy, and as being non-responders if plasma HCV RNA was detectable at end-of-treatment. Patients were classified as having a rapid virological response (RVR) if HCV RNA was undetectable day 29 and a complete early virological response (cEVR) if HCV RNA was undetectable week 12.

### HCV-RNA quantification

Plasma was obtained using PPT-tubes and HCV-RNA was determined by RT-PCR of plasma using Cobas AmpliPrep/COBAS TaqMan HCV Test (Roche Diagnostics, Branchburg, NJ), which quantifies HCV-RNA with a limit of detection of ≤15 IU/mL. HCV-RNA quantification was performed on days 0, 3, 7, 8, 29, week 8, week 12, week 24 (for those receiving 24 weeks of therapy), and 24 weeks after completion of therapy. All samples were frozen (−70°C), and subsequently analyzed at the central laboratory.

### Homeostatic model assessment-insulin resistance (HOMA-IR)

Baseline fasting glucose (mmol/L) was measured locally whereas fasting serum insulin (mU/L, Architect Insulin, Abbott, Abbott Park, IL) was analyzed on frozen samples at the central laboratory. HOMA-IR was calculated using the formula: (Glucose×Insulin)/22.5 [17]. It should be noted that a standardized reference range is lacking for HOMA-IR [18].

### Interferon α2a drug concentration

Plasma concentration of interferon-α2a was measured at day 3, 7 (*i.e.* immediately before the second dose of peginterferon-α2a) and 29. All samples were collected using PPT-tubes, frozen (−70°C), and subsequently analyzed at a central laboratory. Quantification was performed using 20 µl plasma (diluted 1∶5) on a solid-phase ELISA using high absorbant plates coated with interferon alfa antibody (AMS Biotechnology Ltd., Oxon, UK). The detection limit of the assay was 400 pg/mL.

### Ribavirin concentration

Plasma samples for ribavirin drug concentration measurement were drawn immediately before the morning dose of ribavirin at day 29 (week 4) and at week 12, *i.e.* trough concentrations. Plasma ribavirin concentrations were measured by use of solid phase extraction and high-performance liquid chromatography (HPLC; Merck-Hitachi, Tokyo, Japan) followed by UV-detection (wavelength 215 nm).

### Liver biopsies

Liver biopsies were obtained from all patients within 24 months prior to study entry. The evaluation was performed in a blinded fashion by two independent observers according to the Ishak protocol [19]. In addition, steatosis was graded as follows: absent = 0, less than 30% of hepatocytes involved = 1, 30–70% of hepatocytes involved = 2, and more than 70% of hepatocytes involved = 3 [20].

### Statistical methods

Wilcoxon-Mann-Whitney U-test, Kruskal-Wallis test, Chi squared (χ^2^) test, and Spearman's rank correlation coefficient (*r_s_*) test was utilized to evaluate relationships between variables. After univariate analyses, multivariate analyses were performed on the per-protocol patients treated for 24 weeks with all variables associated with the endpoint (P<0.1) being entered. The variables entered in the multivariate analyses were age, BMI, Ishak fibrosis stage, steatosis grade, HOMA score, peg-interferon concentrations on day 3, 7 and 29, ribavirin concentrations day 29 and week 12, and baseline HCV RNA level. All statistical analyses were performed using SPSS (version19.0) and StatView (version 5.0, SAS Institute Inc., Cary, NC, USA). All reported p-values are two-sided, and p-values<0.05 were considered significant.

### Ethical considerations

Written informed consent was obtained from each participating patient. Ethics committees in each participating country approved the study (i.e. Regional Ethical Review Board, Gothenburg, Sweden (Regionala etikprövningsnämnden i Göteborg), Regional Committee for Ethics in Medical Research, Bergen, Norway (Regionaletisk komite for medisinsk og helsefaglig forskning i Bergen), The Scientific Ethical Committee for the Region of Middle Jylland, Viborg, Denmark (Den Videnskabsetiske Komité for Region Midtjylland), The Scientific Ethical Committee for the Region of South Denmark, Vejle, Denmark (Den Videnskabsetiske Komité for Region Syddanmark), and the Ethics Committee, Department of Medicine for the Hospital District of Helsinki and Uusimaa, Finland (Etiska kommittén för invärtesmedicin)). The study has been registered at the NIH trial registry (ClinicalTrials.gov Identifier: NCT00143000).

## Results

**Figure 1:**
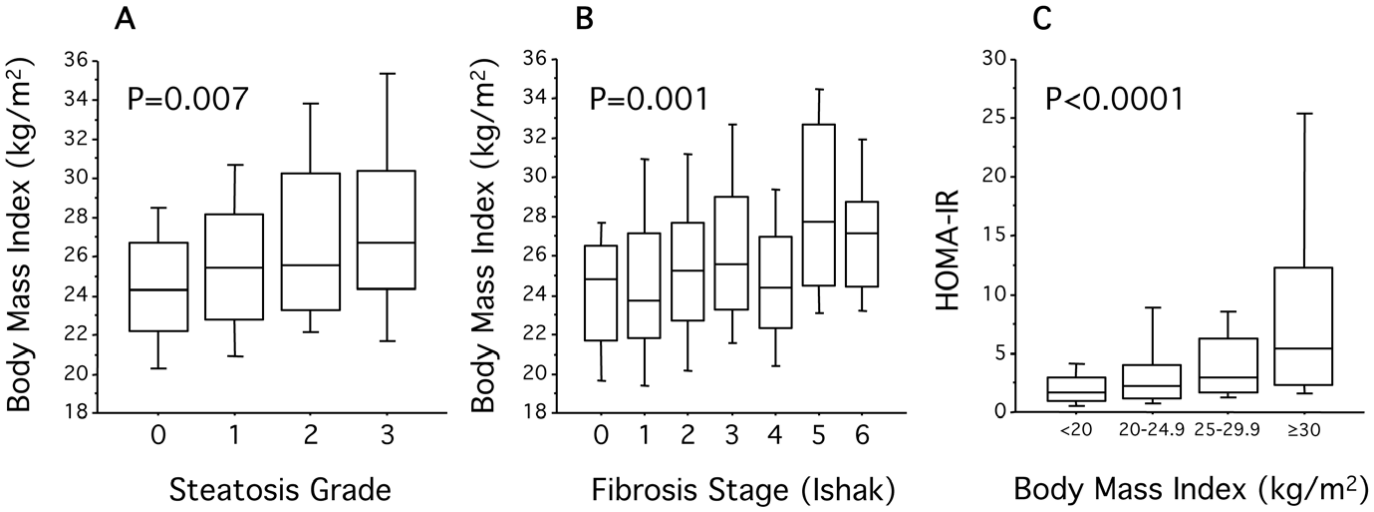
Impact of BMI on baseline histopathology and HOMA-IR. Box plots displaying the 10^th^, 25^th^, 50^th^, 75^th^, and 90^th^ percentiles of BMI among patients grouped by steatosis grade (A; n = 279) and fibrosis stage (B; n = 279), as well as HOMA-IR among patients grouped according to BMI (C; n = 268). P-values obtained using Kruskal-Wallis test.

A strong association was noted between liver histopathology and body mass index (BMI) as well as HOMA-IR with patients having higher BMI also having higher HOMA-IR (r_s_ = 0.31, P<0.0001), indicative of insulin resistance and more pronounced steatosis and fibrosis (Figure 1). Spearman's rank correlation coefficient (r_s_) test for correlation between BMI and HOMA-IR was 0.31 (P<0.0001). When grouping according to HCV genotype, the association between steatosis and BMI remained significant for both genotypes 2 and 3, as did the correlation between BMI and HOMA-IR for genotype 3. As expected, patients with BMI ≥30 kg/m^2^ also had significantly higher systolic and diastolic blood pressure as well as baseline triglycerides levels (mean 1.5 vs. 1.0 mmol/L BMI ≥30 vs. <30 kg/m^2^, P = 0.0002; Table 1) indicative of the metabolic syndrome. In contrast BMI was not associated with IL28B genotype.

**Figure 2:**
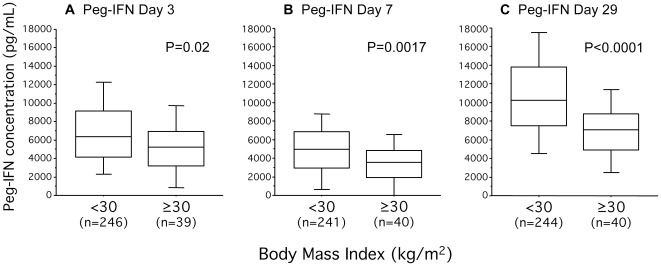
Impact of obesity on interferon concentrations. Box plots displaying the 10^th^, 25^th^, 50^th^, 75^th^, and 90^th^ percentiles of the plasma concentrations of peginterferon (pg/mL) treatment day 3, 7, and 29 (**A–C** respectively). P-values obtained using Mann-Whitney U-test.

**Figure 3:**
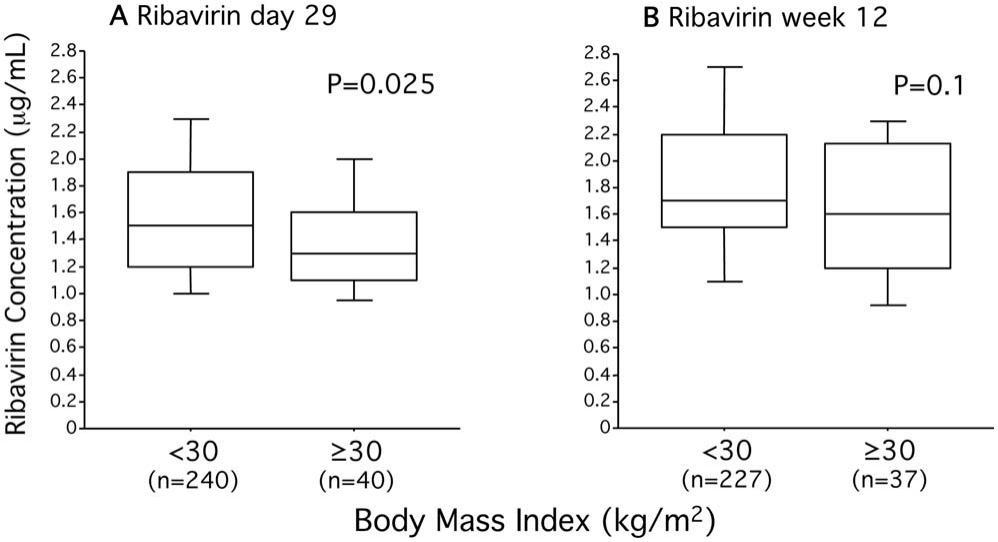
Impact of obesity on ribavirin concentrations. Box plots displaying the 10^th^, 25^th^, 50^th^, 75^th^, and 90^th^ percentiles of the plasma concentrations of ribavirin (µg/mL) treatment day 29 and week 12 (**A** and **B** respectively). P-values obtained using Mann-Whitney U-test.

Higher BMI was correlated with significantly lower concentrations of interferon as measured on days 3 (*r_s_* = −0.21, P = 0.0006), 7 (*r_s_* = −0.28, P<0.0001), and 29 (*r_s_* = −0.38, P<0.0001), (association dichotomized for BMI < or ≥30 kg/m^2^ demonstrated in Figure 2) as well as ribavirin day 29 (*r_s_* = −0.32, P<0.0001) and week 12 (*r_s_* = −0.37, P = 0.0002) (association dichotomized for BMI < or ≥30 kg/m^2^ demonstrated in Figure 3), and these correlations were significant for both HCV genotype 2 and 3. Because the first dose of study medication was administered under the supervision of a study nurse, the lower interferon concentrations noted on days 3 and 7 could not be attributed to poor patient compliance. Similarly subsequent dosing was monitored by a patient diary, and only patients having received at least 80% of the target dose of peginterferon-α2a and at least 80% of the target dose of ribavirin for at least 80% of the target treatment duration were included in the analysis. Thus lower adherence to therapy was unlikely to contribute to the lower concentrations of interferon and ribavirin measured on day 29, and ribavirin at week 12. It should be noted that women overall had approximately 10% higher ribavirin concentrations even when adjusting for weight as previously reported [21], and that this has previously been hypothesized to be secondary to a lower erythrocyte particle concentration noted among women thus allowing for less erythrocyte-trapping of ribavirin.

**Figure 4:**
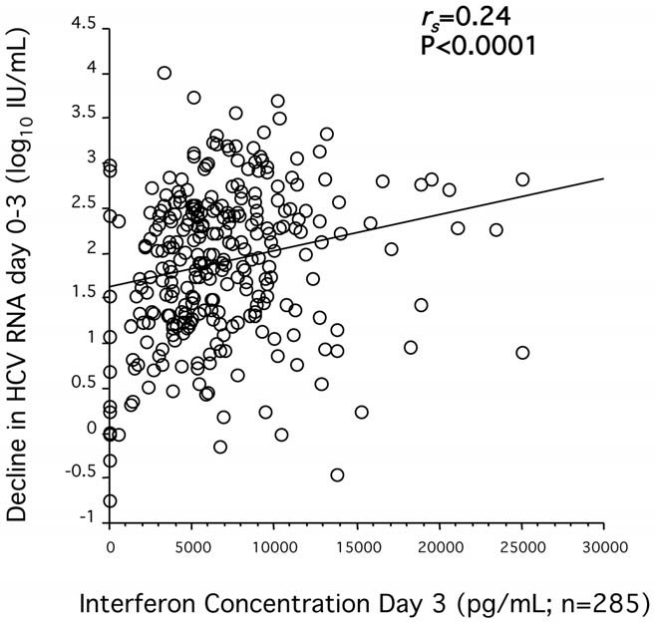
Impact of interferon concentration on first phase decline in HCV RNA. Correlation between the plasma concentration of peginterferon (pg/mL) treatment day 3 and decline in HCV RNA (log_10_ IU/mL) between baseline and treatment day 3 of combination therapy evaluated by use of Spearman's rank correlation coefficient *r_s_* test.

Interferon concentrations at all three time-points sampled were significantly associated with the first phase decline in HCV RNA (Figure 4) for both HCV genotypes 2 and 3, but not with the second phase (r_s_ = −0.05 and P = 0.4, r_s_ = −0.022 and P = 0.7, and r_s_ = 0.005 and P = 0.9 for interferon concentrations on days 3, 7, and 29 respectively); and this association between interferon concentrations and the first phase decline was independent of IL28B genotype. In contrast, ribavirin concentrations were not associated with either the first (r_s_ = 0.018 and P = 0.8 and r_s_ = −0.018 and P = 0.8 for ribavirin concentrations on day 29 and week 12 respectively) or second phase reduction of HCV RNA (r_s_ = 0.018 and P = 0.8 and r_s_ = 0.042 and P = 0.5 for ribavirin concentrations on day 29 and week 12 respectively).

**Figure 5:**
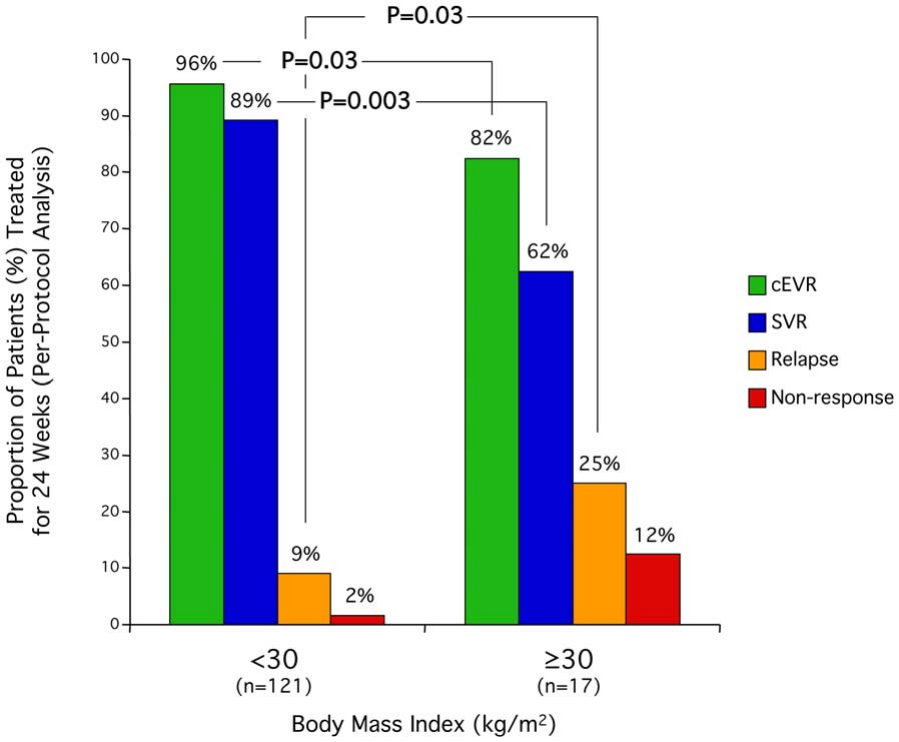
Impact of obesity on outcome. Histogram displaying the proportion achieving cEVR, SVR, relapse or non-response among patients treated for 24 weeks included in the per-protocol analysis grouped according to BMI. P-value obtained using Chi-squared test.

Somewhat surprisingly higher BMI was correlated with higher baseline viral load (r_s_ = 0.12, P = 0.038), and this correlation became more pronounced while on treatment as exemplified by treatment day 3 (r_s_ = 0.18, P = 0.002), day 7 (r_s_ = 0.15, P = 0.01), day 8 (r_s_ = 0.20, P = 0.0006), day 29 (r_s_ = 0.33, P<0.0001), week 8 (r_s_ = 0.45, P<0.0001), and week 12 (r_s_ = 0.49, P<0.0001). Similarly a non-significant trend towards a smaller first phase decline in HCV RNA as measured by the reduction from day 0 to 3 (mean 1.66 vs. 1.91 log_10_ IU/mL BMI ≥30 vs. <30 kg/m^2^) and second phase as measured by the reduction from day 8 to 29 (mean 0.79 vs. 0.83 log_10_ IU/mL per week BMI ≥30 vs. <30 kg/m^2^). However, the association noted between higher BMI and higher HCV RNA levels day 29 was not reflected by the proportion of patients achieving undetectable HCV RNA, i.e. RVR (20% vs. 18%, BMI ≥30 vs. <30 kg/m^2^). In contrast, obese patients were less likely to achieve undetectable HCV RNA week 12 (i.e. cEVR; Figure 5).

In addition to the effect on viral kinetics, BMI profoundly affected SVR especially among patients treated for the standard 24 weeks of treatment duration (Figure 5) with only 62% of patients with BMI ≥30 kg/m^2^ achieving SVR as compared to 89% among those with BMI <30 kg/m^2^ (P = 0.006). This decrease in SVR was mirrored by increased rates of relapse and non-response for both HCV genotypes 2 and 3. In univariate analysis of the population treated for 24 weeks, both peg-interferon and ribavirin concentration day 29 where significantly associated with SVR (P = 0.01), and in a multivariate analysis, ribavirin concentrations week 12, peg-interferon concentrations day 29, and baseline HCV RNA levels were independent predictors of achieving SVR (Table 2). Among patients treated for 12 weeks, those achieving SVR had significantly lower BMI than those that did not (mean 25.5 vs. 27.2 kg/m^2^; P = 0.02).

**Table 2:** Multivariate logistic regression to identify factors predictive of achieving SVR among per-protocol patients treated for 24 weeks.

	Odds Ratio (95% CI)	*P*
Ribavirin concentration week 12 (mg/L)	3.41 (1.03–11.26)	0.04
Peg-Interferon concentration day 29 (ng/mL)	1.22 (1.04–1.44)	0.02
Baseline HCV RNA level (log10 IU/mL)	0.23 (0.08–0.70)	0.01

## Discussion

In spite of the pending introduction of direct antiviral agents (DAA) in routine clinical practice for HCV genotype 1, few of the currently available DAAs are active against genotype 2 or 3. Thus interferon-α and ribavirin are likely to retain pivotal roles in the management of chronic HCV genotype 2/3 infection, and a fixed dose of 800 mg/day ribavirin in conjunction with fixed 180 µg dosing of peginterferon-α2a [6] or weight-adjusted dosing of peginterferon-α2b [22] currently is the standard of care for these viral genotypes. In this setting, the main finding in the present study was that higher BMI was associated with significantly lower concentrations of ribavirin and interferon as well as poorer outcome following fixed dosing of ribavirin and peginterferon-α2a, that lower plasma concentrations of interferon were associated with a poorer first phase reduction in HCV RNA, and that both ribavirin and interferon concentrations along with baseline viral load were independent predictors of SVR.

The lower bioavailability of peginterferon noted in our study corroborates the findings reported by Lam *et al.* (among 11 HCV infected patients, 6 obese and 5 non-obese) who reported a non-significant trend towards lower interferon concentrations and higher baseline viral load following a single dose of 10 mIU of unpegylated interferon-α2b without the addition of ribavirin among obese patients. Interestingly in contrast to the present study, no difference in the first phase decline in HCV RNA was noted though the sample size was small [15].

The finding in the present study that patients with BMI ≥30 kg/m^2^ had significantly lower concentrations of ribavirin following fixed dosing of 800 mg ribavirin/day is not surprising, and may substantiate the report by Pattullo *et al.* that weight based dosing of ribavirin may negate the influence of BMI and weight on outcome [23]. However, this latter study had only a limited number of patients infected with genotypes 2 (n = 22) or 3 (n = 31), and a substantial proportion of patients received weight based dosing of peginterferon-α2b. Fried *et al.* noted that among difficult-to-cure (*i.e.* body weight >85 kg and viral load >800,000 IU/mL) HCV genotype 1 infected patients higher fixed doses of both ribavirin (1600 mg daily) and peginterferon-α2a (270 µg/week) improved viral kinetics and SVR rates as compared to lower doses of ribavirin (1200 mg daily) and peginterferon-α2a (180 µg/week) [24]. This is in contrast to the PROGRESS study where no benefit was noted from giving 1400/1600 mg ribavirin daily as compared to 1200 mg in an intention-to-treat analysis among difficult-to-treat (*i.e.* body weight ≥85 kg and viral load ≥400,000 IU/mL) HCV genotype 1 infected patients [25]. It should be noted, however, that 42% of these patients had a BMI below 30 kg/m^2^, and no subset analysis restricted to patients with BMI ≥30 kg/m^2^ has yet been reported. Additionally in a per-protocol analysis restricted to patients with at least 80% exposure to the planned ribavirin dose, numerically higher SVR rates were obtained with intensified regimens suggesting that such strategies may be successful if tolerated [25].

Similarly it may be hypothesized that patients with BMI ≥30 kg/m^2^ may benefit from increased dosing of interferon, which is substantiated by the abovementioned study by Fried *et al.* [24]. However, in the PROGRESS study no significant differences in SVR were noted in patients weighing ≥85 kg receiving a 12-week induction regime of 360 µg peginterferon-α2a weekly as compared to standard dosing in an intention-to-treat analysis [25]. However, when the analysis was restricted to patients tolerating at least 80% of the planned interferon dosing, numerically higher SVR rates were achieved with intensified regimens reiterating the importance of adherence [25]. Additionally the potential benefit of exposure to higher doses of interferon beyond 12 weeks of therapy was not investigated in this latter study. Similarly, in the IDEAL study, no significant difference in SVR was noted among patients with higher weight regardless whether they received weight based dosing of peginterferon-α2b or a fixed 180 µg dosing of peginterferon-α2a although a non-significant trend towards slightly higher SVR rates was noted in patients weighing between 105 and 125 kg who received weight based dosing of interferon (44.9%, 42.1%, and 38.9% for peginterferon-α2b 1.0 and 1.5 µg/kg body weight and 180 µg peginterferon-α2a respectively) [26]. However, it should be noted that in this weight class patients treated with peginterferon-α2b received 1400 mg ribavirin daily as opposed to 1200 mg daily for patients treated with peginterferon-α2a, and thus lower ribavirin dosing may have contributed to the lower numerical SVR rates noted following fixed interferon dosing.

In addition to differences in bioavailability of interferon and ribavirin, we also noted that patients with higher BMI had higher baseline viral load, more pronounced steatosis, in addition to features of the metabolic syndrome as previously reported, e.g. higher systolic and diastolic blood pressure, higher HOMA-IR, and higher baseline triglyceride levels. Although weight reduction among obese HCV patients is known to normalize many aspects of the metabolic syndrome as well as liver histology [27], [28] and thus is likely to have a beneficial impact on combination therapy for HCV, this cannot be addressed in the present study. Similarly whether increased treatment duration beyond 24 weeks for patients with BMI ≥30 kg/m^2^ would improve outcome is outside the scope of this study. Since replication, assembly and release of HCV occurs in close proximity to intracellular lipid droplets [29], the finding of a higher baseline viral load among obese patients, hypothetically, may be secondary to the elevated pre-treatment plasma triglyceride levels or to more advanced steatosis.

In conclusion, higher BMI is associated with lower bioavailability of both peginterferon and ribavirin, which subsequently along with higher baseline viral load are dominant risk factors for treatment failure in obese patients with chronic HCV genotypes 2 and 3 infection.
